# A systematic review of the economic burden of colorectal cancer

**DOI:** 10.1002/hsr2.70002

**Published:** 2024-08-21

**Authors:** Abdosaleh Jafari, Fatemeh A. Hosseini, Faride S. Jalali

**Affiliations:** ^1^ School of Health Management and Information Sciences, Health Human Resources Research Center Shiraz University of Medical Sciences Shiraz Iran; ^2^ Student Research Committee Shiraz University of Medical Sciences Shiraz Iran

**Keywords:** colorectal cancer, cost, cost of illness, economic burden, review

## Abstract

**Background:**

Colorectal cancer is the third most common cancer in the Western Hemisphere. It is the third most common cancer in men after prostate and lung cancers and the second most common cancer in women after breast cancer. According to some studies, the incidence and prevalence of colorectal cancer is increasing rapidly.

**Main Body:**

In the present study, a systematic review of the articles related to the economic burden of colorectal cancer was carried out. The articles were taken from the following databases: SID, Medline/Pubmed, Embase, Scopus, Web of Science, NHS Economic Evaluation Database (EED), Econlit, and Google Scholar. Furthermore, the PICOTS model was used to select the inclusion criteria. The quality of the articles' methodologies was evaluated using Drummond's checklist. Then, some data were extracted from relevant articles, in terms of year, place of research, sample size, costing approach, type of measured costs, average direct medical costs, average direct nonmedical costs, and average indirect costs. The data from 37 studies dealing with the costs of patients with colorectal cancer were extracted. Most of the studies were conducted in the United States, and the social perspective was the most common perspective to measure the costs. According to the majority of the studies, direct medical costs were considered the greatest driver in causing the economic burden of colorectal cancer. The costs of hospitalization, medicine, surgery, chemotherapy, and radiotherapy accounted for the largest share of direct medical costs, and the costs of transportation, accommodation, and home care were the greatest share of direct nonmedical costs. Furthermore, the costs associated with disability, absenteeism, and premature death were identified as the main drivers of indirect costs.

**Conclusion:**

The findings of this study showed that colorectal cancer imposes great direct and indirect costs on families, the health system, and society. The best way to deal with this disease and, hence, to reduce its economic burden is to take comprehensive preventive measures and modify the lifestyle. In addition, health policymakers can limit the costs of this disease by expanding the screening program.

## 1 BACKGROUND

Cancers are among the diseases of the 20th century that affect more than 14 million people worldwide every year. All cancers are curable if detected early. Cancer cells will continue to grow except in cases such as surgical removal of the cancerous mass, use of chemotherapy or hormone therapy, use of radiation therapy, or sometimes shrinking and disappearing of cancer cells.^1^ Lung cancer and breast cancer are the leading causes of cancer death in men and women, respectively. Colon cancer is also considered a cause of cancer deaths in men and women in developed countries.^2^ It is the third most common cancer in the Western Hemisphere, the third most common cancer in men after prostate and lung cancers, and the second most common cancer in women after breast cancer.^3^ One of the factors affecting colorectal cancer is age; the prevalence of this cancer increases with age, and it can be treated by surgery, radiation therapy, and chemotherapy.^4^ The incidence of colorectal cancer in people under 50 years of age has been increasing rapidly in the last 20 years.^5^ In addition to the aging and eating habits of the populations in high‐income countries, adverse risk factors such as obesity, reduced physical activity, and smoking increase the risk of developing colorectal cancer.^6^ This cancer occurs sporadically in the context of hereditary cancer syndromes or is caused by inflammatory bowel diseases.^7^ The findings obtained from previous studies showed that colorectal tumors had immune responses, and a number of patients with advanced colon cancer achieve long‐term benefits through immunotherapy.^8^

Every year, more than 945,000 people are diagnosed with colon cancer worldwide, and about 492,000 patients die.^7^ The global burden of colon cancer is projected to increase by 60% to more than 2.2 million new cases and 1.1 million deaths by 2030.^9^ Most cases of colon cancer are diagnosed in Western countries, and the incidence rate increases every year.^10^ The prevalence of this cancer is higher in men than in women, and the mean age of diagnosis in developed countries is about 70 years.^11^ A study by Iraqi et al. in 2019 suggested that the overall incidence of colon cancer was declining in many high‐income countries, yet the analyses conducted in the United States and other high‐income countries such as Australia, Canada, and Norway showed an increase in the incidence of the disease in adults under 50 years of age. However, in the most recent 10‐year period for which the data were available, significant increases were observed in the incidence of colorectal cancer in people under 50 years of age in Denmark (3.1%), New Zealand (2.9%), Australia (2.9%), and the United Kingdom (1.8%). A significant increase was also seen in the incidence of rectal cancer in this age group in Canada (3.4%), Australia (2.6%), and England (1.4%). Meanwhile, the incidence of bowel cancer in people aged 50–74 years significantly decreased in Australia (1.6%), Canada (1.9%), and New Zealand (3.4%), and rectal cancer also decreased in Australia.^12^ Studies also showed that 4.3 million new cancer cases and 2.9 million new cancer deaths occurred in China in 2018; these statistics show that the prevalence of cancer in China is lower than in the United States and England, but the mortality from cancer is 30%–40% more.^13^ In Iran, the number of people suffering from this disease in recent years has been reported to be 10,696, and it is increasing as well.^14^ Furthermore, the number of young patients diagnosed with colon cancer has increased for unknown reasons.^15^

The economic burden of cancer on national health expenditures is billions of dollars. The economic costs are measured based on direct and indirect medical costs and vary depending on the stage of diagnosis, the age of the patient, the type of medical services, and the place of the services. The costs vary by region, physician behavior, and patient preferences.^16^ Colorectal cancer cost the EU 126 billion euros in 2009; the cost of cancer healthcare was €102 per citizen across the EU, but it ranged from €16 per person in Bulgaria to €184 per person in Luxembourg.^17^ A study by Jafari et al. in 2019 showed that the mean annual cost for each patient with colon cancer in Iran was $116,917,762.^14^ Colon cancer in the Iranian population starts at a younger age than in Western countries. This imposes a significant direct and indirect economic cost on the community. The direct medical cost of colon cancer in Iran is much more than $38 million.^18^

The present study was conducted to investigate the economic burden of colorectal cancer using a systematic review. The results of this review study, which was conducted for the first time, can be used in cost‐effectiveness and cost‐utility analyses of the diseases associated with colorectal cancer, and they should also be used in the preparation and formulation of clinical guidelines.

## 2 METHODS

In the present study, a systematic review was applied, and the articles related to the economic burden of colorectal cancer were used based on the PICOTS model to select the inclusion criteria. The study population consisted of patients with colorectal cancer; the interventions included all types of clinical interventions to treat the patients with colorectal cancer; the comparators were not limited to specific ones; the outcomes included all direct and indirect costs of the patients with colorectal cancer; the time of the study was determined by the articles published before December 2021; and the study design was based on the costing studies that collected the cost data from colorectal cancer patients. The studies relevant to the research objective were retrieved through the search strategy (Table 1).

**Table 1:** The search strategy of the research.

Search strategy
**Databases:** SID, Medline/PubMed, Embase, Scopus, Web of Science, NHS Economic Evaluation Database (EED), EconLit and Google scholar
**Limits:** up to December 2021, language (English and Persian)
**Strategy:** #1 AND #2 **#1** Cost OR expense OR “economic burden” OR “financial burden” OR “direct medical cost” OR “direct cost” OR “indirect cost” **#2** “colorectal cancer”

**Figure 1:**
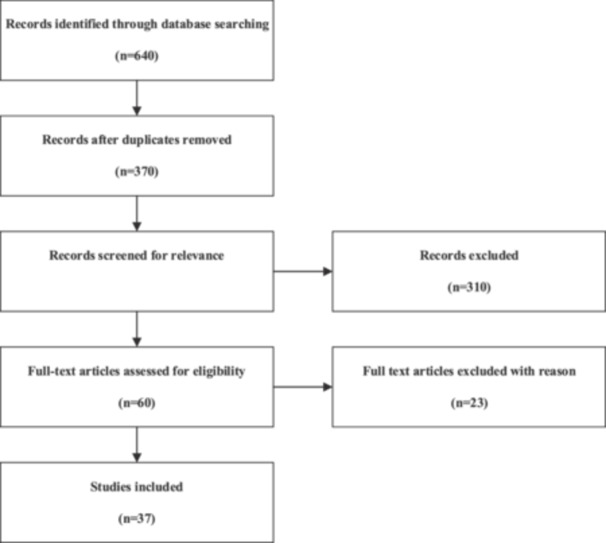
Preferred Reporting Items for Systematic Reviews and Meta‐Analyses (PRISMA) diagram for the systematic review process.

The relevant studies were selected in several steps. First, 640 articles were indexed in all databases. After removing the duplicates, 370 articles were selected to be reviewed, of which 310 were removed from the list after reviewing their titles and abstracts, and 60 others were selected for full‐text review. The Drummond checklist was used to evaluate the quality of the articles' methodologies, and parts of the checklist that were not suitable for conducting costing studies were removed from the checklist.^19^ Finally, the research team selected 37 articles (Figure 1). It is worth mentioning that all research processes and selection of the articles were done by two independent researchers, and the third researcher was responsible for consensus if necessary. In the fourth and last step, a data graph form was used to extract the data from each study. The data of the articles were extracted in terms of year, place of research, sample size, research perspective, costing approach, types of measured costs, and average direct medical, direct nonmedical, and indirect costs. It should be noted that since the studies had been conducted in different countries and in different years, to make comparisons, the costs were updated using the CPI (consumer price index) extracted from the World Bank website.^20^

## 3 RESULTS

After reviewing the articles based on the PICOTS model, the data from 37 studies that investigated the costs of patients with colorectal cancer were extracted. The results of the present review are listed in Tables 2 and 3. Table 2 shows the characteristics of the studies on the costs of colorectal cancer patients based on the years and places of the studies, sample sizes, costing approaches, and the types of costs measured.

**Table 2:** Selected studies on the economic burden of colorectal cancer.

N	Author	Year	Place	Participants	Study perspective	Approach	Measured cost type
1	Emmert(21)	2012	Germany	181	Third‐party payer	‐	Direct costs
2	Fernandez(17)	2013	27 European countries	‐	Societal	Bottom‐up	Direct medical costs, direct nonmedical costs, indirect costs
3	Hanly(22)	2013	Ireland	154	‐	‐	Direct costs and indirect costs
4	Yaldo(23)	2014	America	165	‐	‐	Direct medical costs and indirect costs
5	Mesti(24)	2014	Slovenia	209	Hospital	‐	Direct medical costs
6	Hugo(25)	2015	Europe	33 countries	‐	Bottom‐up	Direct medical costs, direct nonmedical costs, indirect costs
7	Govaert(26)	2015	Netherlands	6782	Hospital	Bottom‐up	Direct costs
8	Hall(27)	2015	Great Britain	145	NHS	Top‐down	Direct costs
9	Inotai(28)	2015	Hungary	8457	‐	‐	Direct medical costs
10	Azzani(29)	2016	Malaysia	138	Participants and families	‐	Direct medical costs, direct nonmedical costs, indirect costs
11	Hanly(30)	2016	Ireland	159	‐	‐	Direct medical costs, direct nonmedical costs, indirect costs
12	Yajima(31)	2016	Japan	30	Third‐party payer	‐	Direct medical costs
13	Marti(32)	2016	United Kingdom	83	Societal	Top‐down	Direct medical costs, direct nonmedical costs, indirect costs
14	Li(33)	2016	China	175	‐	‐	Direct medical costs
15	Sagar(34)	2017	United States	3901	Health care system	‐	Direct medical costs
16	Alefan(35)	2017	North Jordan	97	Business	Bottom‐up	Direct medical costs
17	Liu(36)	2017	China	448	‐	‐	Direct medical costs
18	Johnston(37)	2017	United States	2352	‐	‐	Direct medical costs
19	Rezende(38)	2018	Brazil	234,457	Health care system	‐	Direct costs
20	Goldsbury(39)	2018	Australia	7624	‐	Bottom‐up	Direct costs
21	Gordan(40)	2018		753	‐	‐	Direct medical costs
22	Reyes(41)	2019	America	3707	‐	‐	Direct medical costs
23	Jafari(14)	2019	Iran	96	Participants and families	Bottom‐up, prevalence based	Direct medical costs, direct nonmedical costs, indirect costs
24	Henaine(42)	2019	Lebanon	179	Third‐party payer	‐	Direct medical costs
25	Shi(43)	2019	China	14,536	Business	‐	Direct medical costs
26	Vekic(44)	2019	Serbia	21,000	Societal	‐	Direct medical costs
27	Yin(45)	2019	China	515,615	‐	‐	Direct medical costs
28	Yezefsk(46)	2020	America & Canada	2494	‐	‐	Direct medical costs
29	Wu(47)	2020	Beijing	1026	‐	‐	Direct medical costs
30	Mittmann(48)	2020	Canada	38,108	Third‐party payer	Bottom‐up	Direct medical costs
31	Justiano(49)	2020	New York State	12,218	‐	Bottom‐up	Direct medical costs
32	Huang(50)	2020	Taiwan	112,986	Societal	Bottom‐up	Direct medical costs, direct nonmedical costs, indirect costs
33	Ibarrondo(51)	2021	Basque	7801	Health care system	‐	Direct medical costs
34	Pattamatta(52)	2021	Netherlands	184	Societal	Bottom‐up, prevalence based	Direct medical costs, direct nonmedical costs, indirect costs
35	Nejati(53)	2021	Iran	657	Health care system	‐	Direct medical costs, direct nonmedical costs, indirect costs
36	Pasmans(54)	2021	Netherlands	24 laboratories	‐	Bottom‐up	Capital and current expenses
37	Mulder(55)	2021	Netherlands	155	Societal	Bottom‐up, prevalence based	Direct medical costs, direct nonmedical costs, indirect costs

**Table 3:** Average direct and indirect costs associated with the economic burden of colorectal cancer (2021 USD).

N	First author	Direct medical costs			Direct nonmedical costs			Indirect costs			Total cost	
		Per patient (mean)	Total	Most constituents	Per patient (mean)	Total	Most constituents	Per patient (mean)	Total	Most constituents	Per patient (mean)	Total
1	Emmert(21)	1316.1	238,326.6	Cytostatics drugs	1032.4	186,880	Transportation	‐	‐	‐	2349	425,317
2	fernandez(17)										12.58	14,980,000
3	Hanly(22)	1092.7	168,271.9	Hospital‐related costs	344.9	53,126.6	Transportation	1769.1	272,460.5	‐	2396	221,650,000
4	Yaldo(23)	6132.07	1,010,209.2	‐	‐	‐	‐	352.77	58,205.9	Short‐term disability	‐	‐
5	Mesti(24)	22,231.91	22,231.9	Systematic therapy	‐	‐	‐	‐	‐	‐	‐	‐
6	Hugo(25)		8,244,000	Hospital care		1,099,200	‐	‐	1,758,000	Loss of productivity due to disability	‐	11,101,200
7	govaert(26)	17,478.69	1,179,193,505	Ward	‐	‐	‐	‐	‐	‐	18,129.02	1,223,110,592
8	Hall(27)	13,030.13	1,889,370.09	Hospitalization	‐	‐	‐	‐	‐	‐	‐	‐
9	Inotai(28)	2277.87	‐	Inpatient cost	‐	‐	‐	‐	‐	‐	3753.42	‐
10	Azzani(29)	13,089.99		Surgery	861.1		Travel	1577.23			15,496.53	
11	Hanly(30)	‐	‐	‐	‐	‐	‐	8870.18	1,398,989.20	Temporary disability	‐	‐
12	Yajima(31)	‐	6305.74	Anticancer drugs								26,924.47
13	Marti(32)										6102.28	504,876.9
14	Li(33)										1856.98	924,067.35
15	Sagar(34)										94,529.85	
16	Alefan(35)	7928.3	765,168.8	Drug cost								
17	Liu(36)	1907.16		Western medicine								
18	Johnston(37)	3408.84										
19	Rezende(38)	2565.5	601,115,235.8	Chemotherapy								
20	goldsbury(39)										1271.14	
21	gordan(40)	18,457.8									18,698.4	
22	Reyes(41)			Office visit costs	‐	‐	‐	‐	‐	‐	17,405.94	
23	Jafari(14)	11,001.536	223,125,000	Surgeries	2511.52	49,215,000	Accommodation	1264.35	25,750,000	Patient companions' absenteeism due to patient care	14,650.49	298,009,000
24	Henaine(42)										355,021.63	
25	Shi(43)	9034.89		Surgery								
26	Vekic(44)	2223.47	46,814,724	Radiotherapy								
27	Yin(45)	3780.2	272.83	inpatient cost								
28	Yezefsk(46)										5294.64	
29	Wu(47)	8636.61		Surgery								
30	Mittmann(48)	6839		Radiation								
31	Justiano(49)	24,842.65										
32	Huang(50)	12,358		Medication				11,954.18		Mortality	24,312.12	
33	Ibarrondo(51)			Chemotherapy							20,023.71	
34	Pattamatta(52)	1841.22		Hospital stay	1354.4		Paid homecare	176.38		Inability to do Unpaid labor	3370.26	
35	Nejati(53)	23,001.3	10,728,220.5	Inpatient drug	5923.8	2,767,366.35		‐	‐	‐	28,926.45	13,495,586.85
36	Pasmans(54)	364.67		Diagnostics								
37	Mulder(55)	891.38		Nursing	126.09		Informal care	11.45		Absenteeism in paid work	1028.92	

As observed in Table 2, the largest number of studies had been conducted in the United States. The largest sample size was 515,615 people, and the social perspective was the most common perspective for measuring the costs. In addition, 15 of the 37 studies (40.54%) had not mentioned their perspectives, and one study had been conducted from the perspective of the patient, hospital, and a third party. The common costing approach had been the population‐based approach, and 23 studies (62.16%) had not mentioned their costing approaches. The most measured costs were direct medical costs, and nine studies measured both direct and indirect costs.

According to Table 3, 25 studies (69.44%) had mentioned direct medical costs as the most important driver of the economic burden on patients with colorectal cancer, and only one study (2.77%) mentioned indirect costs as the main driver of this economic burden. Among the studies calculating direct medical costs (*n* = 25), nine studies (36%) had mentioned hospitalization costs as the greatest part of this type of cost, while five (20%), four (16%), four (16%), and three (12%) studies had reported drug costs, surgery costs, chemotherapy and radiotherapy costs, and other costs as the major components of direct medical costs, respectively. Of the six studies dealing with the components of direct nonmedical costs, four studies (66.66%) reported travel and accommodation costs, and two studies (33.34%) reported the cost of home care as the most important component of direct nonmedical costs. Furthermore, seven studies addressed the components of indirect costs, of which three studies (42.87%), three studies (42.87%), and one study (14.26%) mentioned the costs associated with disability, absenteeism, and premature death as the main drivers of indirect costs, respectively.

According to Table 3, the highest and the lowest mean direct medical costs were found in the studies conducted in the New York state, United States, (USD 24,842.65) and in the Netherlands (USD 364.67), respectively. On the other hand, the highest and the lowest mean direct nonmedical costs were reported in Iran at $5923.8 (USD) and in the Netherlands at $126.09 (USD), respectively. Regarding indirect costs, the highest average cost was reported in Taiwan (USD 11,954.18), and the lowest was found in the Netherlands (USD 11.45).

## 4 DISCUSSION

The incidence of colorectal cancer (CRC) around the world is high and is mainly related to the countries' economic development levels. The cost‐of‐illness studies provide information on the economic burden of diseases, which is useful in setting priorities for the development of health programs. In addition, it may lead to financial support for health service users and equitable distribution of health services.^21^ Therefore, the aim of this study was to estimate the economic burden of CRC through the use of a systematic review.

The direct costs imposed on the health system, society, families, and patients include medical and nonmedical costs. The former refers to medical care expenses for diagnosis, treatment, and rehabilitation.^18^ Direct medical costs were the main drivers of the economic burden of colorectal cancer patients in the studies reviewed in this research;^14^, ^22^, ^23^, ^24^, ^25^, ^26^, ^27^ among their components, hospital costs^28^, ^29^ and drug costs were the highest. Färkkilä (2015) stated in his study that CRC brought about significant costs, which varied significantly in different stages of the disease. Direct costs are the highest within the first 6 months after diagnosis due to initial treatment, including surgical intervention and hospitalization^30^; thus, hospital costs can be considered as one of the main drivers of direct medical costs.

The studies by Alefan et al. (2017) in Jordan^31^ and Davari (2012) in Iran^32^ reported that drug costs were the main cost factor for patients with colorectal cancer in all stages. In 2020, Huang in Taiwan found out that the reason for the high cost of medicine was the increased demand for anticancer drugs.^25^ In their study, Leigh et al. (2021) also pointed out the high cost of anticancer drugs and attributed it to factors such as the great demand and willingness to pay due to the lethality and high risk of this disease, high research and development costs, and the failure of market forces.^30^

The results of the present research showed that the most important drivers of direct nonmedical costs were the expenses related to travel and accommodation of the patients and their companions^14^, ^24^, ^28^ as well as home care expenses.^27^, ^33^ In their study, Jafari et al. (2019) stated that some of the main reasons for the high cost of travel and accommodation for the patients and their companions were the lack of related medical centers in the cities where they lived, the long distance to the treatment and diagnostic centers, and the postponement of chemotherapy due to the lack of unoccupied beds and, as a result, the patient's need for long stay in inns and hotels.^14^ According to Hanly et al. in Ireland, the reason for the high cost of transportation in their country was the high transportation tariff.^28^

In this study, the greatest indirect costs were associated with disability,^23^, ^29^, ^34^ absenteeism,^14^, ^27^, ^33^ and premature death,^25^ respectively. Yaldo et al. (2014) stated in research that since most of the patients with colorectal cancer were of working age, the average costs related to disability and absence from work were high.^22^ As stated by Jafari et al. (2019), the cost of absenteeism was high because when referring to hospitals and healthcare centers, each patient was usually accompanied by more than one person who worked for organizations. For this reason, the cost of the patient's companions' absenteeism due to accompanying and taking care of the patient increased.^14^

It was found in this study that the highest average direct medical, direct nonmedical, and indirect costs had been reported in New York state (USA), Iran, and Taiwan, respectively, and the lowest costs had been reported in the Netherlands. In addition to different tariffs of services in different studies and countries, different research perspectives could also be the reason for the difference in costs. Besides, the difference in the demographic characteristics of the studied patients (including age and gender), the stage of the disease, and even the limitation of a number of studies to the estimation of some cost components could be considered among the causes of the difference in direct and indirect costs. Complicating factors such as the characteristics of health care systems, payer models, and data availability vary across countries.^32^ Around the world, total cost estimates and patient levels vary widely, making accurate international comparisons of costs nearly impossible. Therefore, it is better to define a specific standard for reporting cost‐of‐illness studies, including the standardization of explicit determination of patient populations, types of medical services, and selection of methods. Furthermore, the design of such studies should reflect a thorough understanding of the health system's payment and reimbursement policies and their impact on the available data to enable comparisons between costs. Many studies have only examined some part of the costs of colorectal cancer patients and have neglected important costs such as reduced productivity as well as the cost of lost time for the patients and their companions. Thus, paying attention to these cost components should be on the agenda of the researchers in this field in the future. It should be noted that creating a more comprehensive picture of the economic burden of cancers for patients and families could help them make better decisions in their jobs. It may also be important for employers and politicians interested in minimizing the impact of cancer on the job of the patient and his/her companions, including attendance or absenteeism, productivity at work, and overall maintenance of their job positions.

This study had several limitations, including the limitations related to the databases and search strategies used by the researchers.

## 5 CONCLUSION

The results of this research showed that most of the studies on the economic burden had a social perspective, and direct medical costs were recognized as the greatest driver of the economic burden of colorectal cancer. Among the components of direct medical costs were the costs of hospitalization, medicine, surgery, chemotherapy, and radiotherapy. In addition, some of the components of direct nonmedical costs were the costs associated with travel, accommodation, and home care, and indirect costs included the costs associated with disability, absenteeism, and premature death as the main drivers. The present study also provided evidence on the estimation of direct and indirect costs of the patients with colorectal cancer, indicating the high economic burden of this disease for the patients and their families, the payers (insurance), and the health system. This economic burden is predicted to increase significantly due to the increased number of newly diagnosed cases; this necessitates the implementation of prevention and screening programs in the National Cancer Control Program or at least in the treatment of patients in the early stages of the disease. Suggesting general recommendations for diet and lifestyle modification can also reduce the risk of CRC. Replacing red and processed meat, and sugars with fish, chicken, vegetables, fruits, and grains may reduce the risk of developing this neoplasm. In addition, avoiding smoking and alcohol consumption, and reducing obesity also appear to be beneficial.

Future studies could take advantage of the reported data, focusing on the costs of inpatient and outpatient care and their predictors in patients with colorectal cancer, to ensure appropriate resource allocation in the future.

## AUTHOR CONTRIBUTIONS

Fatemeh A. Hosseini and Faride S. Jalali did the search screening and data extraction. Abdosaleh Jafari raised the research idea and supervised all phases of the research. All authors have equal contributions in drafting and reviewing the manuscript. All authors have read and approved the final manuscript.

## CONFLICT OF INTEREST STATEMENT

The authors declare no conflict of interest.

## ETHICS APPROVAL AND CONSENT TO PARTICIPATE

All procedures performed in studies were in accordance with the ethical standards. Under the code of IR. SUMS. NUMIMG. REC.1401 by the Shiraz University of Medical Sciences.

## TRANSPARENCY STATEMENT

The lead author, Faride Sadat Jalali, affirms that this manuscript is an honest, accurate, and transparent account of the study being reported; no important aspects of the study have been omitted; and any discrepancies from the study as planned (and, if relevant, registered) have been explained.

## Data Availability

Data from this research is available and could be sent upon contact with the corresponding author.
